# Left Atrial Function Post Radiofrequency and Cryoballoon Ablation Assessed by Volume-Pressure Loops

**DOI:** 10.3389/fcvm.2022.830055

**Published:** 2022-03-09

**Authors:** Antonios Karanasos, Konstantinos Tyrovolas, Dimitrios Tsiachris, Michalis Efremidis, Athanasios Kordalis, Maria Karmpalioti, Efstathia Prappa, Stefanos Karagiannis, Constantina Aggeli, Konstantinos Gatzoulis, Dimitrios Tousoulis, Costas Tsioufis, Konstantinos P. Toutouzas

**Affiliations:** ^1^1st Department of Cardiology, Athens Medical School, Hippokration Hospital, Athens, Greece; ^2^Second Department of Cardiology, “Evangelismos” General Hospital of Athens, Athens, Greece; ^3^Athens Heart Centre, Athens Medical Centre, Athens, Greece

**Keywords:** catheter ablation—atrial fibrillation, atrial fibrillation, left atrium, real-time three-dimensional echocardiography, volume pressure loops

## Abstract

**Background:**

Left atrial (LA) function is linked to atrial fibrillation (AF) pathogenesis. AF catheter ablation decreases disease burden with potentially favorable effects on cardiac function. Atrial volume-pressure loops can optimally assess the LA function.

**Objective:**

To investigate changes in LA function by volume-pressure loops after paroxysmal AF ablation and explored potential differences between the radiofrequency and cryoballoon ablation.

**Methods:**

We analyzed 44 patients undergoing paroxysmal AF ablation from 2 centers, 22 treated with radiofrequency and 22 with cryoablation. Pre- and post-procedure, all patients underwent a real-time three-dimensional transthoracic ECG to evaluate LA volume, while simultaneously recording LA pressure following transseptal puncture. Volume-pressure loops pre- and post-procedure were created by paired data. Areas of A-loop (LA booster pump function) and V-loop (LA reservoir function), and the stiffness constant determining the slope of the exponential curve during LA filling were calculated.

**Results:**

Average LA pressure, A-wave amplitude, and V-wave amplitude were increased post-procedurally (*p* < 0.001). Overall, A-loop area decreased (*p* = 0.001) and V-loop area tended to increase (*p* = 0.07). The change in both A-loop and V-loop areas was similar between radiofrequency- and cryoballoon-treated patients (*p* = 0.18 and *p* = 0.52, respectively). However, compared with cryoballoon-treated patients, radiofrequency-treated patients had higher increase in the stiffness constant (*b* = 0.059; 95% CI: 0.022–0.096; *p* = 0.006).

**Conclusion:**

AF catheter ablation by the radiofrequency or cryoballoon is associated with the decrease of the booster pump function and increase of the reservoir function. Moreover, there is a post-procedural increase of LA pressure which is associated with an acute increase in LA stiffness in radiofrequency ablation, but not in cryoablation.

## Introduction

Atrial fibrillation (AF) is associated with significant patient morbidity and mortality (1). Recent research has led to the consideration of AF as a manifestation of atrial cardiomyopathy (2). This is supported by observations linking left atrial (LA) anatomy and physiology to pathogenesis of AF, with a prominent role of LA remodeling and fibrosis, while increased LA pressures play important roles in the initiation and recurrence of this arrhythmia (3). Catheter ablation of AF by pulmonary vein (PV) antral isolation and PV-LA junction ablation is an established therapy that decreases the disease burden, thus leading to reverse remodeling, decreasing LA size, and improving left ventricular (LV) function (4, 5). Yet, the mechanisms by which these actions are mediated have not been completely elucidated, while several studies have demonstrated controversial results regarding its impact on LA function, with stiff LA syndrome described in a small percentage of patients following LA ablation (6).

Atrial volume-pressure loops are the optimal method to assess LA function, based on the classic Newtonian mechanics (2, 7, 8). The loops allow physiological assessment of atrial systolic function, by providing information on LA work during reservoir and contraction phases, as well as on LA stiffness (8). Changes in atrial function induced by catheter ablation of AF have not been documented thus far using this approach.

The aim of this prospective study was (1) to investigate potential changes in LA function by volume-pressure loop assessment after catheter ablation of AF by PV antral isolation and PV-LA junction ablation in patients with paroxysmal atrial fibrillation (PAF), and (2) to explore a potential difference in this response between the radiofrequency catheter and cryoballoon ablation.

## Materials and Methods

### Study Population

This is a prospective observational study of patients undergoing catheter ablation of PAF. Consecutive patients over 18 years old with PAF undergoing for the first time catheter ablation of PAF by PV antral isolation and PV-LA junction ablation, either with a radiofrequency or a cryoballoon catheter, as per clinical indication, were enrolled from 2 high-volume centers from 1 December 2017 to 31 October 2018. Each center enrolled consecutive patients undergoing PV antral isolation using the same method; i.e., radiofrequency ablation for “Evangelismos” General Hospital and cryoablation for Athens Medical Center. All patients included were required to have ≥2 paroxysms of PAF defined as self-terminating AF paroxysms or cardioverted within 7 days, were on sinus rhythm at the time of the ablation, and maintained sinus rhythm immediately after the procedure. Patients with severe mitral stenosis or regurgitation, previous cardiac surgery, any prosthetic valve, evidence of ischemia, LA thrombus, or severe LV systolic or diastolic dysfunction were excluded. Additionally, patients with echocardiographic images of suboptimal quality (i.e., inability to image the left atrium during the entire cardiac cycle, or very low frame rate not allowing a real-time 3D echocardiography analysis) or poor quality ECG recordings were excluded from the *post-hoc* analysis.

### Study Protocol

Patients underwent PV isolation either by a radiofrequency or a cryoballoon catheter, according to the institutional practice and local standards, as described in the “Ablation Procedure” section. Before and after the procedure, all patients underwent a real-time 3D transthoracic ECG to evaluate LA volume changes during an entire cardiac cycle, while simultaneously recording LA pressure following the transseptal puncture. After the procedure, LA volume and pressure recordings were gated offline by ECG, and recorded values were used to plot the LA pressure as a function of LA volume. Demographic, clinical, and 2d-echocardiographic variables were prospectively obtained. All patients gave written informed consent. The protocol was approved by the institutional ethics committees and complied with the Declaration of Helsinki.

### Ablation Procedure

All procedures were performed under conscious sedation according to institutional practices, either by radiofrequency ablation or cryoablation (9, 10). Conscious sedation for both methods included administration of intravenous boli of midazolam, while intravenous fentanyl was administered as bolus for analgesia. Heparin was administered before the transseptal puncture and maintained at activated clotting time levels >300. Briefly, for radiofrequency ablation, following a single transseptal puncture, LA was reconstructed by the CARTO 3 navigation system (Biosense Webster, CA, USA) and wide circumferential lesions around both ipsilateral PVs were performed by a 3.5 mm-tip ablation catheter (Thermo Cool Navi-Star and Smart Touch, Biosense Webster) and a power setting of 30–40 W. The endpoint of the ablation was the absence or dissociation of potentials in the isolated area as documented by the circular mapping catheter (Lasso, Biosense Webster) (10).

Cryoablation was performed without the 3D mapping. A 15-F steerable sheath (FlexCath, Medtronic, Minneapolis, Minnesota) was used to introduce the cryoballoon catheter system into the LA. A 28 mm cryoballoon (Arctic Front Advance, Medtronic) was then advanced through the FlexCath sheath, and positioned at the antrum of each PV guided by the circular mapping catheter (Achieve, Medtronic). The ablation strategy was wide antral isolation and additional LA lesions were not allowed. The protocol recommended the use of 240 s cryoapplications using a freeze-thaw-freeze technique. An additional freeze was delivered in the case of failure to isolate the PV, if time to isolation was >60 s, and in cases where a mapping catheter was unable to monitor the real-time isolation. To minimize the risk of phrenic nerve injury, right phrenic nerve pacing was performed by an electrode catheter in the superior vena cava and capture was confirmed by palpation and intermittent fluoroscopy. Application of cryoenergy was terminated immediately upon attenuation or loss of phrenic nerve capture.

For both procedures, entrance and exit block of the PVs were evaluated 30 min after the initial isolation. The procedure was considered complete once all PVs with conduction recovery were re-isolated. In cases where AF occurred during the procedure, when not terminated by ablation, sinus rhythm was restored by cardioversion.

### Transthoracic ECG

**Figure 1:**
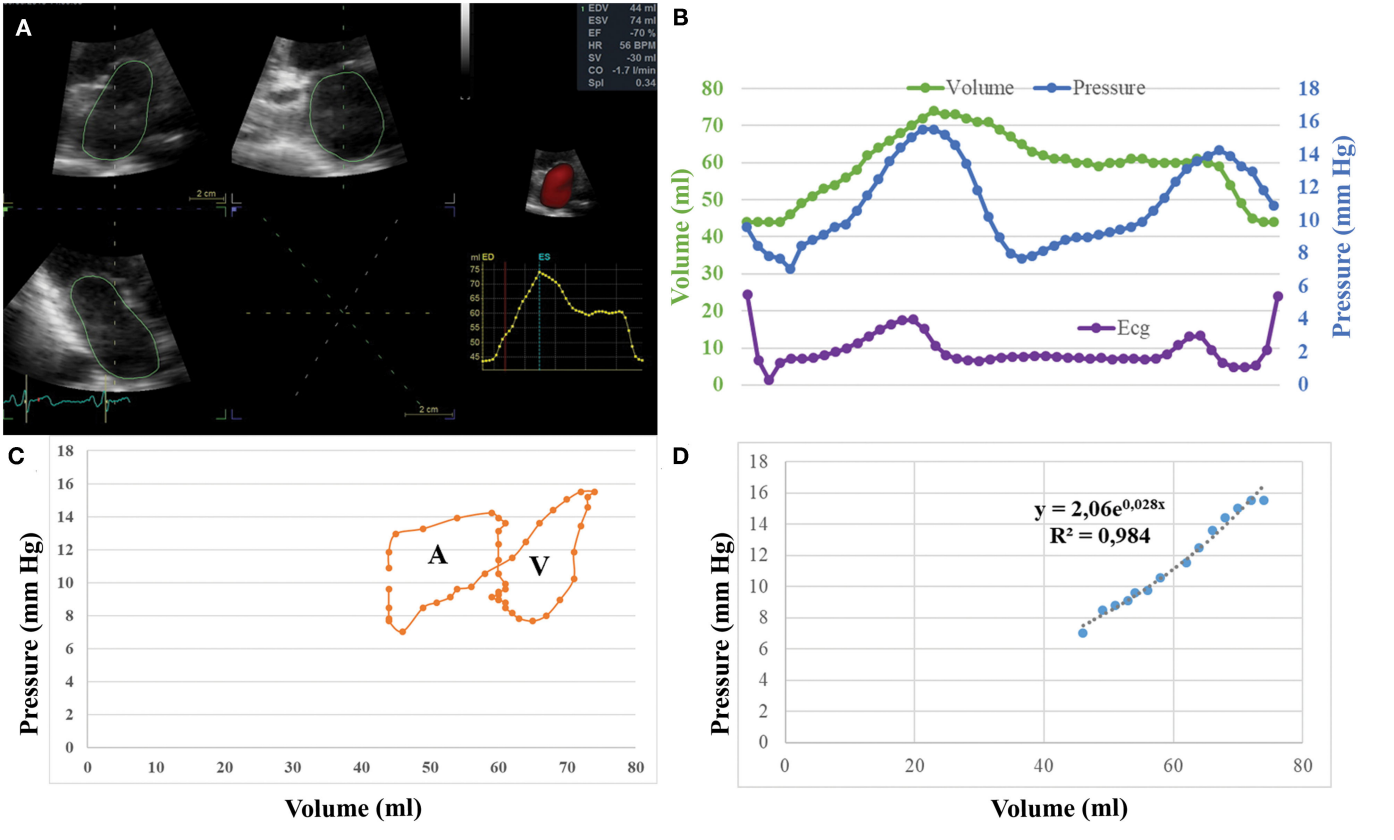
**(A)** Methodology of echocardiographic analysis for left atrial (LA) volume calculation. **(B)** Simultaneous recording of LA pressure and LA volume during a cardiac cycle gated by ECG. **(C)** Graphical plotting of LA volume vs. LA pressure and creation of volume pressure loops. **(D)** Calculation of the passive elastic chamber stiffness constant (cm^−3^) and of the elastic constant (mmHg).

The studies were performed by two experienced echocardiographers (EP, SK) using a Vivid-E9 system with a 4 V probe (GE Healthcare, Chicago, US) at two time points: pre- and post-ablation. By real-time 3D echocardiography, apical full-volume datasets, taking care to avoid foreshortening, were recorded during end-expiratory apnoea. After the procedure, images were analyzed offline at the independent echocardiographic laboratory of Hippokration Hospital, blinded to the ablation method and timing of acquisition. Images were analyzed by two observers (AKa, MK) with a consensus approach, and images were re-evaluated by a third reviewer (CA) to ensure contingency of the analysis, with disagreements resolved by consensus. The LA borders were traced at multiple planes during the cardiac cycle using a semiautomated contour tracing algorithm (4DQ analysis) of the Echopac software (GE healthcare), excluding the PVs and the LA appendage from the volume measurement (11, 12). Thereby, LA volume within a cardiac cycle was calculated and plotted as a function of time with a simultaneous ECG recording (Figure 1). In the case of suboptimal images to reliably calculate LA volume during the systole, the patient was excluded from the study. Moreover, end-diastolic LA volume (LAEDV), end-systolic LA volume (LAESV), and LA volume pre-contraction were recorded, while *total atrial emptying volume* was calculated as LAESV minus LAEDV and *active atrial emptying volume* as LA volume pre-contraction minus LAEDV (12). *Atrial ejection* fraction was calculated as total atrial emptying volume divided by LAESV and multiplied by 100, while *LA active ejection fraction* as active atrial emptying volume divided by LAESV and multiplied by 100 (12).

### LA Pressure Measurement

Following the transseptal puncture, LA pressure was measured by an oscillometric device (Truwave; Edwards Lifesciences) connected to a fluid-filled 6F multipurpose angiographic catheter or to the cryoballoon catheter after removing the Achieve circular mapping catheter. This measurement was performed after transseptal puncture and LA catheterization, just before the ablation procedure, and repeated before the sheath withdrawal. No chronotropic or inotropic agents were administered during measurements. Measurements from a single beat were used for each volume-pressure loop pre- and post-ablation, at an end-expiratory phase and with stable sinus rhythm for at least three beats. All pressure, electrogram, and surface ECG measurements were recorded by the Labsystem Pro software (Boston Scientific), and exported to digital format, containing synchronous values of ECG amplitude and instantaneous pressure at 1 ms intervals.

### Calculation of Indices of LA Systolic Function

Recorded values of volume and pressure were gated offline using the ECG recordings, after adjusting for ultrasound signal delay due to the imaging depth (8). Thereby, paired values of volume and pressure were available. Since the sampling ratio of LA volume values is lower than the pressure sampling, and dependent on the sampling rate of the echocardiographic recording, the number of pairs was defined by the number of LA volume measurements, with a minimum of 12 pairs used. These paired values were used to graphically plot LA pressure as a function of LA volume, thus creating a volume-pressure loop (Figure 1) (8). The A-loop area or LA stroke work index, is a measure of the LA booster pump function. The V-loop area expresses the LA reservoir function. Pressure and volume data during the period of the clockwise ascending limb of the volume-pressure loop were fitted to the exponential function P = ***b*** × e^***a***^·V, where P = instantaneous LA pressure and V = LA volume (2, 13). The least squares method was used for calculation of ***a*** and ***b***, where ***a*** is the passive elastic chamber stiffness constant (ml^−1^) that determines the slope of the exponential curve and quantifies LA stiffness, while ***b*** is the elastic constant (mmHg) representing baseline LA pressure conditions.

### Statistical Methods

All analyses were performed with SPSS 25.0. As this is a pilot study, no official study sample was calculated *ad hoc*. Continuous variables are presented as mean ± SD or as median [interquartile range, IQR], as appropriate, and nominal variables as *n* (%). Normality was assessed by the Shapiro–Wilk test. Between-patient normally distributed variables were compared by *t*-test, while non-normally distributed variables by Mann–Whitney test. Nominal variables were compared by Fisher's exact test. As most echocardiographic, haemodynamic, and volume-pressure loop variables were non-normally distributed, a Generalized Estimating Equation with repeated effects was used to assess the impact of the ablation procedure and of the applied method on these variables. The value of *p* <0.05 indicated statistical significance.

## Results

**Figure 2:**
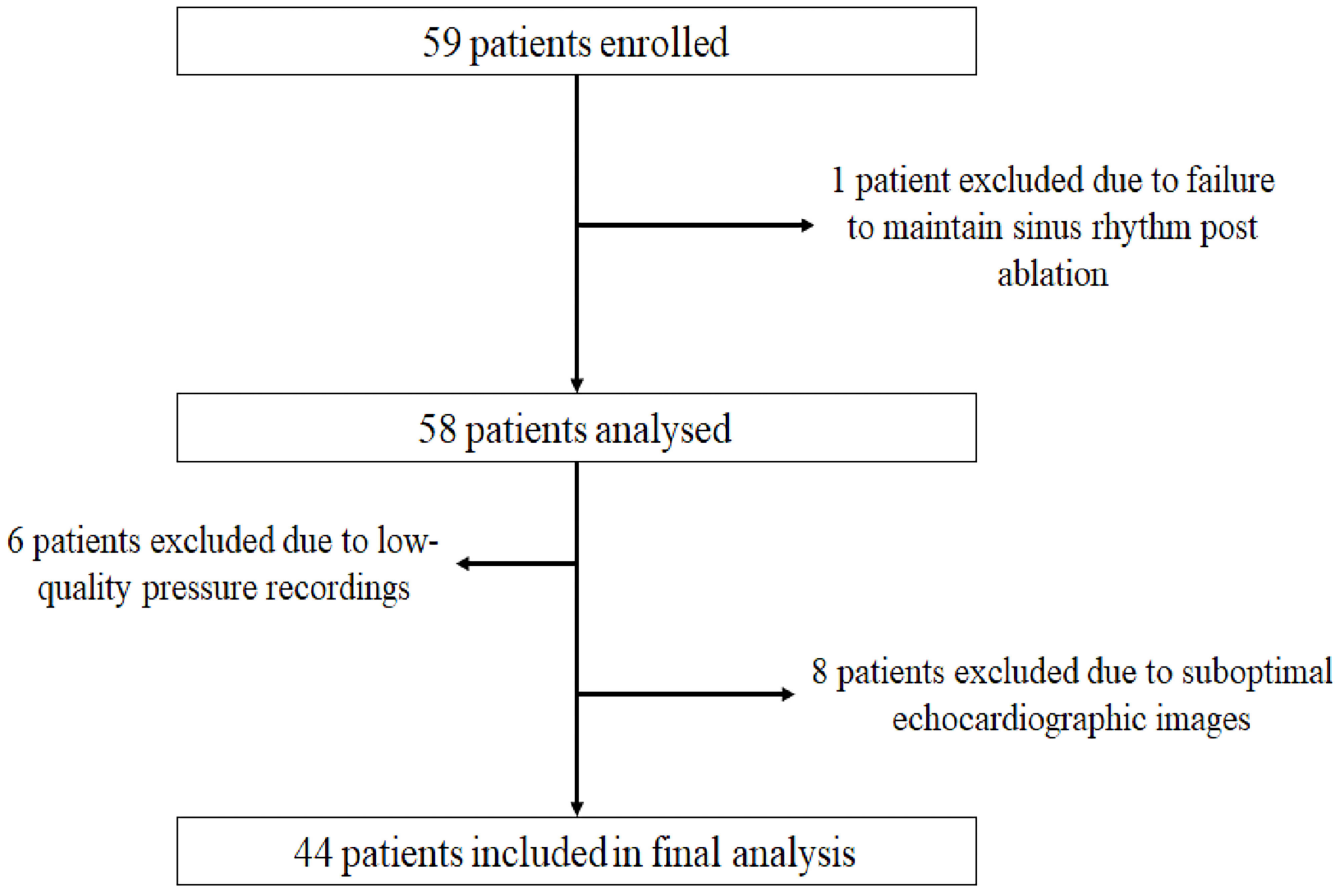
Study flow diagram.

We enrolled 59 patients with PAF. The study flow diagram is presented in Figure 2. Fifteen patients were excluded, eight due to suboptimal echocardiographic images, six due to poor-quality pressure recordings, and one because of failure to convert to sinus rhythm after the end of the procedure. Eventually, 44 patients were used for the analysis, 22 treated with radiofrequency ablation and 22 with cryoablation. The procedure was uneventful in all cases.

### Baseline Characteristics

The mean age was 62.2 ± 10.7 years and 31 patients (70.5%) were men. There were no significant differences in baseline characteristics between patients treated with radiofrequency or cryoballoon (Table 1).

**Table 1:** Patient and ECG characteristics by ablation method.

	**Radiofrequency ablation** **(*n =* 22)**	**Cryoballoon ablation** **(*n =* 22)**	***P*-value**
Age (years)	61.1 ± 9.7	62.3 ± 12.3	0.75
Male gender, n (%)	18(91.7)	13(59.1)	0.19
Hypertension, n (%)	10(45.5)	10(45.5)	0.72
Dyslipidaemia, n (%)	6(27.3)	4(18.2)	0.68
Diabetes mellitus, n (%)	2(9.1)	1(4.5)	0.99
Thyroid disorders, n (%)	2(9.1)	2(9.1)	0.99
Medications			
B-blockers, n (%)	10(45.5)	11(50.0)	0.99
Sotalol, n (%)	1(4.5)	0	0.99
Amiodarone, n (%)	3(13.6)	3(13.6)	0.54
ACE^†^ inhibitors, n (%)	8(36.4)	10(45.5)	0.76
Statins, n (%)	6(27.3)	5(22.7)	0.99
P wave duration pre (msec)	101 ± 15	105 ± 17	0.41
PR interval pre (msec)	173 ± 22	186 ± 29	0.09
QRS interval pre (msec)	88 [80–115]	89[79–106]	0.76
P wave duration post (msec)	103 ± 17	94 ± 21	0.14
PR interval post (msec)	177 ± 27	179 ± 34	0.84
QRS interval post (msec)	93[78–115]	93[79–106]	0.67

^†^*ACE, Angiotensin-converting enzyme*.

### Echocardiographic Analysis

Table 2 summarizes differences before and after the procedure in LA morphometry by real-time 3D echocardiography. There were no significant differences in LAEDV or LA ejection fraction pre- and post-procedure. Conversely, LAESV, total atrial emptying volume, LA volume pre-atrial contraction, active atrial emptying volume, and LA active ejection fraction were significantly lower post-procedurally.

**Table 2:** Procedure-induced changes in echocardiographic, haemodynamic, and volume-pressure variables.

	**Pre**	**Post**	***P*-value^**#**^**
**LA**^**†**^ **volume measurements**			
LAEDV^‡^ (ml)	26.5 [22–38.8]	25.5 [20.3–37.5]	0.68
LAESV^§^ (ml)	59.6 ± 14.9	56.6 ± 15.0	0.04
LAV^¶^ pre-contraction (ml)	41.0 [33.0–50.0]	36.0 [28.0–50.0]	0.006
Total atrial emptying volume (ml)	29.0 [22.0-34.8]	27.0 [22.0–31.0]	0.04
Active atrial emptying volume (ml)	13.0 [8.0–17.0]	8.0 [5.0–10.0]	<0.001
LA ejection fraction (%)	50.2 ± 12.4	48.6 ± 10.4	0.34
LA active ejection fraction (%)	20.5 [16.4–24.6]	14.6 [11.0–18.9]	0.001
**Pressure measurements**			
V-wave (mmHg)	19.4 [15.0–28.6]	24.6 [19.8–34.4]	<0.001
A-wave (mmHg)	16.8 [13.0–23.3]	20.0 [14.3–25.6]	0.001
Average pressure (mmHg)	12.2 [10.0–19.3]	17.3 [12.9–23.8]	<0.001
**Volume-pressure analysis**			
A-loop area (ml*mmHg)	65.7 [32.7–93.7]	39.0 [15.4–67.5]	<0.001
V-loop area (ml*mmHg)	79.5 [32.7–150.2]	92.6 [48.0–181.5]	0.072
Passive elastic chamber stiffness constant (ml^−1^)	0.047 [0.028–0.073]	0.040 [0.021–0.068]	0.63
Elastic constant (mmHg)	1.91 [0.48–4.64]	4.15 [1.75–10.88]	<0.001

*^†^LA: left atrial. ‡LAEDV: left atrial end-diastolic volume. ^§^LAESV: left atrial end-systolic volume. ^¶^LAV: left atrial volume. ^#^Derived from Generalized Estimating Equation*.

Table 3 demonstrates differences in echocardiographic variables between radiofrequency and cryoablation. There were no significant differences between the two groups, except for post-procedural active atrial emptying volume that was higher in patients treated with cryoballoon. Similarly, only active atrial emptying volume and LA volume pre-atrial contraction trended toward higher increase in patients treated with cryoballoon (*p* = 0.05 and *p* = 0.10, respectively), while all other changes pre- and post-procedure in real-time 3D-echocardiographic variables were not significantly different between patients treated with radiofrequency or cryoballoon.

**Table 3:** Echocardiographic characteristics by ablation method.

	**Radiofrequency ablation (*n =* 22)**	**Cryoballoon ablation (*n =* 22)**	***P*-value**
**Baseline TTE findings**			
LVEDD^†^ (mm)	48 ± 3	49 ± 4	0.47
E-wave velocity (cm/s)	80 [60–90]	82 [75–91]	0.37
A-wave velocity (cm/s)	62 ± 16	61 ± 0	0.88
E/A ratio	1.33 [1.13–1.31]	1.28 [1.19–1.62]	0.86
E' Velocity (cm/s)	9 [7–10]	10 [9–11]	0.08
E/E' ratio	9.2 ± 1.6	8.9 ± 3	0.75
**Real-time 3D-echocardiography measurements pre ablation**				
LAEDV^‡^ pre (ml)	26.0 [19.0–39.0]	27.0 [22.0–39.0]	0.61
LAESV^§^ pre (ml)	58.9 ± 14.4	60.2 ± 15.8	0.78
LAV^¶^ pre-contraction pre (ml)	37.5 [32.0–49.3]	44.0 [35.0–54.5]	0.40
Total atrial emptying volume pre (ml)	29.5 [23.8–33.0]	29 [21.8–39.0]	0.97
Active atrial emptying volume pre (ml)	12.5 [7.8–16.0]	14.0 [8.0–17.0]	0.51
LA^#^ ejection fraction pre (%)	51.4 ± 12.4	49.0 ± 12.5	0.53
LA^#^ active ejection fraction pre (%)	21.2 [14.3–24.5]	20.5 [16.5–25.7]	0.88
**Real-time 3D-echocardiography measurements post ablation**				
LAEDV^‡^ post (ml)	24.5 [20.0–35.3]	29.5 [20.5–40.3]	0.47
LAESV^§^ post (ml)	55.9 ± 13.7	57.2 ± 16.5	0.78
LAV^¶^ pre-contraction post (ml)	32.5 [27.8–41.0]	40.0 [30.5–54.0]	0.06
Total atrial emptying volume post (ml)	27.0 [22.0–31.0]	25.5 [20.0–31.8]	0.44
Active atrial emptying volume post (ml)	7.0 [5.0–8.3]	10.0 [7.0–12.5]	0.02
LA^#^ ejection fraction post (%)	50.4 ± 10.2	46.8 ± 10.5	0.25
LA^#^ active ejection fraction post (%)	13.6 [8.9–16.4]	14.7 [11.8–22.5]	0.14

*^†^LVEDD: left ventricular end-diastolic diameter.*

*^‡^LAEDV: left atrial end-diastolic volume.*

*^§^LAESV: left atrial end-systolic volume.*

*^¶^LAV: left atrial volume.*

*^#^LA: left atrial*.

### Haemodynamic and Volume-Pressure Loop Analysis

Table 2 summarizes procedure-induced changes in LA pressure and volume-pressure loop variables. Average LA pressure, A-wave amplitude, and V-wave amplitude were all significantly increased post-procedurally (*p* < 0.001 for all). A-loop area decreased (*p* = 0.001), whereas V-loop area tended to increase (*p* = 0.07). Although the elastic constant increased (*p* < 0.001), there was no significant difference in the passive chamber elastic constant overall (*p* = 0.63).

**Figure 3:**
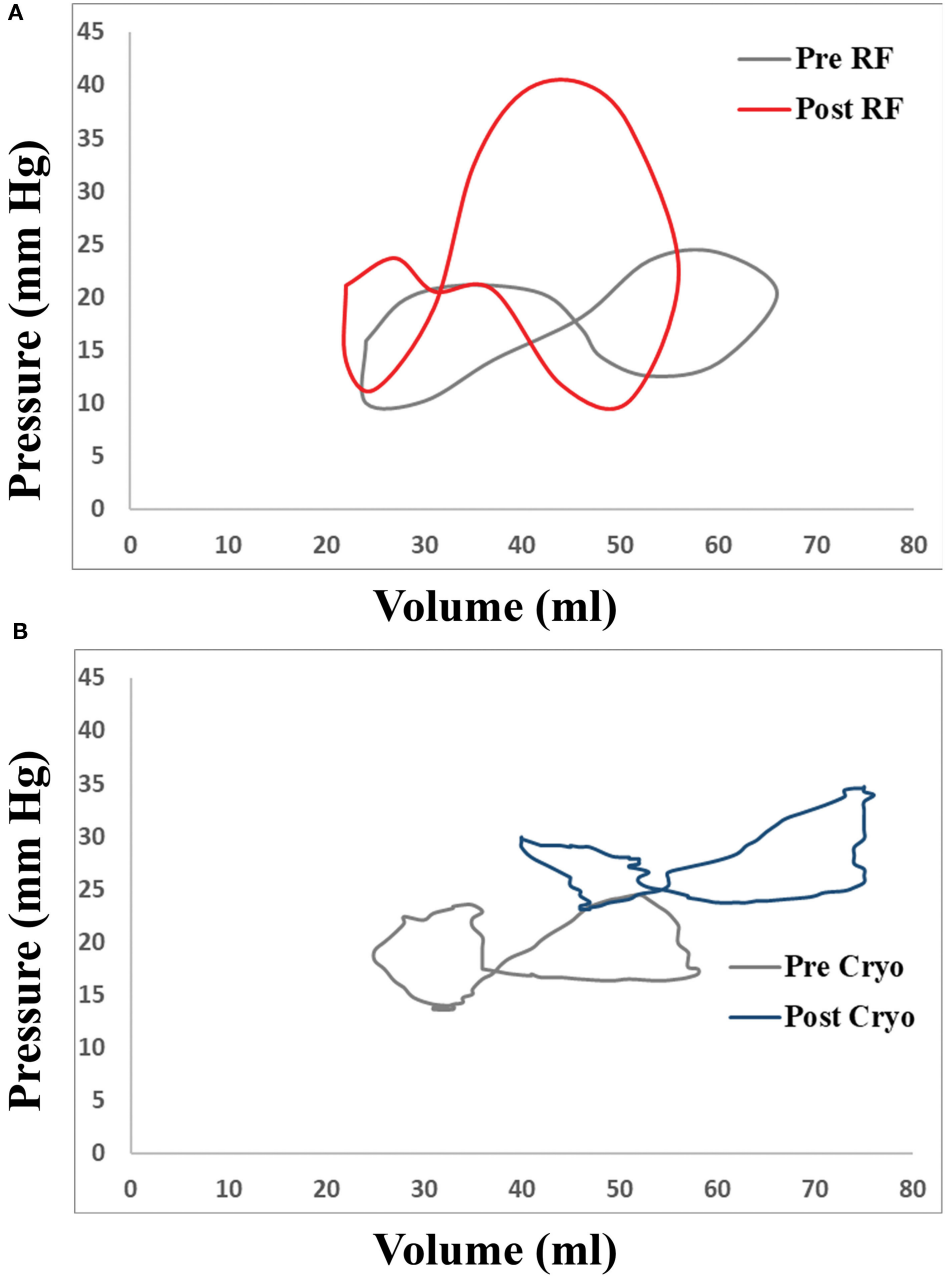
Representative examples of changes in LA volume-pressure loops in patients undergoing transcatheter atrial fibrillation (AF) ablation with **(A)** radiofrequency catheter and **(B)** cryoballoon.

Differences in haemodynamic and volume-pressure loop variables between radiofrequency- and cryoballoon-treated patients are demonstrated in Table 4. Post-procedurally, V-wave amplitude was similar in the two groups (24.3 mmHg [19.1–32.2] vs. 28.1 mmHg [19.9–43.2]; *p* = 0.35), but A-wave amplitude tended to be lower in radiofrequency-treated patients (19.0 mmHg [12.4–22.4] vs. 20.7 mmHg [17.2–37.9], *p* = 0.06), and average pressure was lower (16.6 mmHg [10.4–20.4] vs. 18.7 mmHg [16.5–30.6], *p* = 0.03). There were no significant differences in A-loop or V-loop area between the two groups, although both tended to be higher in radiofrequency-treated patients (A-loop: 40.3 ml^*^mmHg [28.0–70.6] vs. 22.7 ml^*^mmHg [9.5–56.1], *p* = 0.10; V-loop: 112.9 ml^*^mmHg [69.8-198.8] vs. 66.0 ml^*^mmHg [31.4–176.4], *p* = 0.14]. Change in V-wave amplitude was not significantly different between the two groups (*p* = 0.24), whereas increase in A-wave amplitude was lower in radiofrequency-treated patients compared with cryoballoon-treated patients (b = −7.87; 95% *CI*: −14.42 to −1.33; *p* = 0.044) and so was the change in average pressure (b = −8.51; 95% *CI*: −15.00 to −2.02; *p* = 0.021). The extent of change in both A-loop and V-loop areas was similar between radiofrequency- and cryoballoon-treated patients (*p* = 0.18 and *p* = 0.52, respectively). However, compared with cryoballoon-treated patients, radiofrequency-treated patients had lower increase in the elastic constant (b = −6.60; 95% *CI*: −10.56 to −2.64; *p* = 0.005), and higher increase in the passive elastic chamber stiffness constant (b = 0.059; 95% *CI*: 0.022–0.096; *p* = 0.006). To assess a potential impact of peri-procedural fluid administration, especially with the irrigated radiofrequency catheter, we investigated the potential association of total fluid volume, procedural duration, and radiofrequency duration with all post-procedural volume-pressure variables (A- and V-loop area, elastic constant, and passive elastic stiffness constant; Supplementary Table). However, there was no significant correlation for any variable (all *p* > 0.15). Representative examples of volume-pressure loops in patients with radiofrequency and cryoballoon ablation are presented in Figure 3.

**Table 4:** Pressure data and volume-pressure variables by ablation method.

	**Radiofrequency ablation (*n =* 22)**	**Cryoballoon ablation (*n =* 22)**	***P*-value**
**Pressure measurements pre**			
V-wave pre (mmHg)	19.8 [16.2–25.9]	18.9 [13.4–32.9]	0.93
A-wave pre (mmHg)	16.8 [14.0–21.0]	16.8 [11.1–28.8]	0.61
Average pressure pre (mmHg)	12.0 [10.6–15.9]	12.4 [8.5–22.4]	0.41
**Pressure measurements post**			
V-wave post (mmHg)	24.3 [19.1–32.2]	28.1 [19.9–43.2]	0.35
A-wave post (mmHg)	19.0 [12.4–22.4]	20.7 [17.2–37.9]	0.06
Average pressure post (mmHg)	16.6 [10.4–20.4]	18.7 [16.5–30.6]	0.03
**Volume-pressure analysis pre**			
A-loop area pre (ml*mmHg)	71.2 [55.6–114.7]	46.2 [26.9–86.2]	0.08
V-loop area pre (ml*mmHg)	87.1 [61.2–141.0]	73.1 [25.7–152.3]	0.29
Passive elastic chamber stiffness constant pre (ml^−1^)	0.053 [0.038–0.073]	0.031 [0.023–0.081]	0.02
Elastic constant pre (mmHg)	1.07 [0.47–2.40]	3.59 [0.46–5.37]	0.06
**Volume-pressure analysis post**			
A-loop area post (ml*mmHg)	40.3 [28.0–70.6]	22.7 [9.5–56.1]	0.10
V-loop area post (ml*mmHg)	112.9 [69.8–198.8]	66.0 [31.4–176.4]	0.14
Passive elastic chamber stiffness constant post (ml^−1^)	0.063 [0.037–0.100]	0.027 [0.015–0.042]	<0.001
Elastic constant post (mmHg)	2.46 [0.59–4.54]	6.79 [2.74–13.95]	0.001

## Discussion

In this prospective pilot study, we thoroughly investigated the acute impact of PV antral isolation and LA-PV junction ablation on morphometric and haemodynamic metrics of LA function, as well as on metrics derived by volume-pressure loops which can quantify the work of the LA, during both the reservoir and active contraction phases, and the LA stiffness. Moreover, we investigated a possible effect of the applied energy source (i.e., radiofrequency versus cryoballoon) on these changes. The main findings of the present study are that: (1) there is a significant increase of average pressure, A-wave amplitude and V-wave amplitude after the procedure observed in both radiofrequency- and cryoballoon-treated patients; (2) although the total volume emptied from LA to LV does not change post-procedurally, the volume ejected during atrial systole is reduced; (3) both in radiofrequency- and cryoballoon-treated patients, there is a decrease in the LA booster pump function and trend for increase in the reservoir function; and (4) in radiofrequency-treated patients, the increase in LA pressure is mainly due to acute increase in LA stiffness, whereas in cryoballoon-treated patients, this increase is observed without such change in LA stiffness.

AF ablation by PV antral isolation and PV-LA junction ablation is a procedure that decreases the disease burden and improves AF-free survival (1). Several approaches, such as echocardiography including LA strain assessment, haemodynamic measurements, and volume-pressure relationships have studied the interaction of LA function with catheter ablation of AF. A decreased reservoir function that recovers following successful AF ablation has been demonstrated by atrial strain measurement (14). Similarly, an increase of the LA ejection fraction by the 3D echocardiography after 3 months has been observed after catheter ablation, due to increase of the volume emptied to LV during the reservoir phase. This effect was more pronounced with cryoablation, as compared with radiofrequency ablation (15). This might be explained by our study, where radiofrequency but not cryoballoon ablation was associated with increased LA stiffness post-procedurally.

Moreover, LA hemodynamics have a potential impact on ablation procedure outcome. The increased pre-procedural LA pressure has been associated with adverse LA remodeling and AF recurrence post radiofrequency catheter ablation (3), while vice-versa the procedure itself has been associated with increase of LA pressure over time in patients undergoing redo procedures (16). Additionally, increased LA stiffness (assessed by the pressure difference within a cardiac cycle) was associated with the reduced diastolic function at follow-up of the same patients (16). This has further been corroborated by a study combining MRI imaging of the LA with subsequent invasive LA pressure measurements pre-ablation to generate volume-pressure loops. This study assessed the stiffness by calculating the linear slope of ΔP/ΔV during the diastolic LA filling phase—albeit not taking into account the non-linear correlation between these variables—and found that increased pre-procedural LA stiffness was independently associated with AF recurrence after LA ablation (16). Finally, pre-existing LA fibrosis has been associated with recurrences after PV isolation, further supporting a potential adverse impact of LA stiffness (17).

Nevertheless, no study has used an integrated approach to characterize the acute impact of the ablation procedure on LA function. While the main determinants of the LA reservoir function are the LA myocardial compliance and the LV longitudinal displacement, LA contraction is to a greater extent dependent on ventricular filling pressures (18). In our study, despite a higher A-wave amplitude post-ablation, total LA work during active contraction (A-loop area) was reduced due to a lower active emptying volume, lending evidence to a hypothesis of a “stunned” LA unable to forward the blood into the LV despite a higher filling pressure. However, this acute adverse impact of the ablation procedure is counteracted by a compensatory increase of the reservoir function that manages to maintain an unchanged total emptying volume and atrial ejection fraction due to the increase in LA filling pressures.

In our study, both radiofrequency and cryoablation resulted in similar changes in LA function and hemodynamics: reservoir function was increased and booster pump function was decreased in the same extent, while average LA pressure was similarly increased in both methods. This increase in pressure might share common mechanisms in the two groups, such as change of preload with periprocedural fluid administration, or a potential impact of increased vagal stimulation, given the increased passive emptying and reduction of booster function. However, the increase in LA pressure for patients treated with radiofrequency ablation was clearly associated with a higher increase in LA stiffness, as assessed by the passive elastic chamber stiffness constant, which determines the slope in the exponential equation that expresses the changes in LA pressure as a function of volume (2, 13). A similar increase in LA stiffness has been described in an experimental model of the Cox maze procedure (19). It is unclear whether this differential response in LA stiffness in our study might be explained by differences in myocardial injury between the methods. In radiofrequency ablation, effective lesions are transmural and characterized by permanent electrical changes ensuring PV isolation, whereas cryo-lesions are characterized by preserved tissue architecture and cause less damage to the endocardium (20). These histologically well-demarcated and homogeneous lesions might be associated with a subtler response which does not increase LA stiffness to the same extent.

### Perspectives

Using an integrated approach, we documented several important changes underlying catheter ablation of AF. Namely, we observed an acute decrease in active atrial emptying volume with a preservation of the total volume, an acute decrease of the LA booster pump function and an increase of the reservoir function, increase in LA pressures, and in radiofrequency ablation increased LA stiffness. These changes were observed in varying extents among individual patients, and future studies need to examine whether these changes can be used as surrogate markers for assessing the long-term success of the procedure, and whether they might translate to changes in diastolic function over long term, given the well-defined relationship between LA physiology and diastolic filling of the LV (7).

### Limitations

Although the study was not randomized, we included consecutive patients from two different hospitals with each hospital performing a single AF ablation method. Thus, there were no significant baseline differences between the groups. Moreover, the analysis was blinded to the method applied. An *ad-hoc* study sample was not predefined, and thus our study might be underpowered for endpoints with high variability, such as echocardiographic measurements. However, we used a real-time three-dimensional echocardiographic approach which improves reproducibility of the measurements and thus reduces bias (11). As we excluded patients with severe systolic or diastolic dysfunction or severe mitral valve dysfunction, our findings do not necessarily apply to such populations. The exclusion of patients with poor quality echocardiographic or pressure data limited our study sample, but enhanced the robustness of our data. Although micromanometers are the golden standard for invasive pressure measurements, fluid-filled systems remain the mainstay of invasive pressure measurement, as they also have high reproducibility and sampling rate, clearly superior to the sampling rate of echocardiographic measurements. Moreover, the same method was used pre- and post- procedurally, while poor quality tracings were excluded. Additional imaging modalities could have further corroborated the results of our study. Cardiac magnetic resonance is the standard for morphometric evaluation of left atrium with fast strain-encoded sequence allowing the evaluation of strain, while it may delineate myocardial scar within the left atrium, a surrogate marker of reduced compliance and atrial cardiomyopathy (4, 21). Additionally, a 3D echocardiography derived LA strain could have assisted in a more comprehensive evaluation of LA function (22). Nevertheless, the pressure-volume assessment remains an ideal tool for the acute assessment of physiological changes in a research setting, although not easily applicable in everyday practice (2). Non-invasive follow-up by the above mentioned methods will help investigate the long-term implications of the findings of the current study.

## Conclusions

Catheter ablation of AF either by radiofrequency or cryoballoon is associated with functional changes of the left atrium, with a decrease of the booster pump function and an increase in the reservoir function. Moreover, there is a post-procedural increase in LA pressures which in radiofrequency ablation is associated with an acute increase in LA stiffness, but not in cryoablation. Further studies are needed to elucidate the significance of this change on the patient outcome and the long-term diastolic function.

## Data Availability Statement

The raw data supporting the conclusions of this article will be made available by the authors, without undue reservation.

## Ethics Statement

The studies involving human participants were reviewed and approved by Ethics Committee of Hippokration Hospital. The patients/participants provided their written informed consent to participate in this study.

## Author Contributions

AKa: conception of the design, drafting of the manuscript, and data analysis. KT, DTs, ME, and AKo: data analysis and revising the manuscript. MK, EP, SK, and CA: data analysis. DTo, KG, and CT: revising the manuscript. KPT: conception of the idea and design, revising the manuscript, and final approval. All authors contributed to the article and approved the submitted version.

## Conflict of Interest

The authors declare that the research was conducted in the absence of any commercial or financial relationships that could be construed as a potential conflict of interest.

## Publisher's Note

All claims expressed in this article are solely those of the authors and do not necessarily represent those of their affiliated organizations, or those of the publisher, the editors and the reviewers. Any product that may be evaluated in this article, or claim that may be made by its manufacturer, is not guaranteed or endorsed by the publisher.
