# Supplementary material for: Left Atrial Function Post Radiofrequency and Cryoballoon Ablation Assessed by Volume-Pressure Loops
Source: Front Cardiovasc Med. 2022 Mar 9;9:830055. doi: 10.3389/fcvm.2022.830055 (PMC8959489; doi:10.3389/fcvm.2022.830055)
Supplement: Supplementary file 1 [file Table_1.DOCX]

**Supplementary Table.** Regression coefficients of procedural variables for the prediction of post-ablation hemodynamics.

|  | A-loop area post (ml*mmHg) | V-loop area post (ml*mmHg) | Elastic constant post (ml^-1^) | Passive elastic stiffness constant post (mmHg) |
| --- | --- | --- | --- | --- |
| Fluid volume (ml) | -0.099  (-0.220 – 0.021) | -0.207  (-0.578 – 0.165) | -0.009  (-0.040 – 0.023) | 1*10^-4^  (-4*10^-4^ – 2*10^-4^) |
| Procedural duration (min) | -0.098  (-0.522 – 0.326) | 0.282  (-0.986 – 1.550) | -0.013  (-0.120 – 0.093) | -0.001  (-0.002 – 0.0004) |
| Radiofrequency duration (min) | - 1.250  (-9.280 – 6.780) | -11.701  (-30.103 – 6.700) | 0.056  (-1.948 – 2.061) | -0.004  (-0.022 – 0.014) |

All values expressed as b (95% confidence intervals).
